# How to address vaccine hesitancy? Lessons from National Hepatitis B Immunization Program in China

**DOI:** 10.3389/fpubh.2024.1286801

**Published:** 2024-01-22

**Authors:** Haiting Jiang, Chengyu Wei

**Affiliations:** ^1^Department of Medical History and Medical Philosophy, School of Health Humanities, Peking University, Beijing, China; ^2^Department of Epidemiology and Biostatistics, School of Public Health, Peking University, Beijing, China

**Keywords:** vaccine hesitancy, hepatitis B, National Immunization Program, vaccination, decision-making, China

## Abstract

China, with the severe burden of hepatitis B, plays a significant role in the global efforts towards eliminating hepatitis B disease by 2030. Vaccination is recognized as the most effective measure to prevent infectious diseases. However, vaccine hesitancy remains a significant barrier to achieving herd immunity across diverse populations. To address this issue, the health ministries and public health authorities in China have implemented various measures to encourage hepatitis B vaccination. China’s National Hepatitis B Immunization Program, initiated in 1985, has been successful in controlling this vaccine-preventable disease. Given the challenges in eliminating hepatitis B, strengthening the National Hepatitis Immunization Program in China is of utmost importance. Through an analysis of policy documents, reports, and scientific papers, the history of the program was summarized, and effective approaches to address vaccine hesitancy were identified. This will help achieve universal health coverage of vaccines and effectively work towards meeting the goals set for 2030.

## 1 Introduction

China has been classified as a medium to high endemic area for hepatitis B. The burden of hepatitis B virus (HBV) infection is the highest in the World Health Organization (WHO) Western Pacific Region and the WHO African Region (1, 2). HBV has been highly prevalent in China, as evidenced by serosurveys conducted in 1979 and 1992, which indicated a 10% prevalence of HBV surface antigen (HBsAg) (3, 4), with nearly 90 million people living with chronic HBV infection (5), and more than 300,000 deaths caused by HBV infection, accounting for almost half of HBV-related deaths worldwide (6, 7). The United Nations Sustainable Development Goals (SDGs) for 2030 include combating hepatitis and WHO developed a global plan for hepatitis B elimination by 2030. The first *Global Health Sector Strategy on Viral Hepatitis 2016–2021* was published, outlining various goals and targets. One of the quantitative global targets outlined in the strategy is to achieve a 90% reduction in new cases of chronic viral hepatitis B and C infections by the year 2030, along with a 65% decrease in related deaths (2), This means that efforts should be focused on implementing comprehensive preventive measures, diagnosis, and treatment strategies in achieving significant reductions in both new cases and related deaths, such as vaccination campaigns and safe injection practices, to significantly decrease the number of new infections occurring worldwide.

As per the global strategy and to reduce its burden on individuals and communities worldwide, the elimination of hepatitis B disease necessitates the collaborative implementation of five fundamental interventions. These interventions include ensuring widespread vaccination against hepatitis B to protect individuals from acquiring the infection; implementing measures to prevent the transmission of the HBV from infected mothers to their newborns, such as administering antiviral medications and promoting safe delivery practices; enhancing safety measures in healthcare settings to prevent the transmission of hepatitis B through contaminated blood transfusions or unsafe injection practices; providing comprehensive harm reduction services, including access to clean needles and syringes, to reduce the risk of hepatitis B transmission among individuals who inject drugs; and expanding efforts to increase the availability and accessibility of testing and treatment services for hepatitis B, ensuring early diagnosis and appropriate management of infected individuals (2).

The hepatitis B vaccine (HepB) is a safe and effective way to prevent hepatitis B infection, which is typically administered shortly after birth, followed by boosters a few weeks later. It provides nearly 100% long-term protection against the virus. Despite the firmly established evidence showing the immense value of vaccines in preventing disease and disabilities and saving millions of children every year, vaccine hesitancy has now become a growing public concern and attention point (8, 9), which can have a catastrophic impact on public health. When people refuse or delay vaccinations, it can lead to outbreaks of preventable diseases.

Recognizing that vaccine hesitancy is a global challenge, and given its potential to impact on vaccine coverage, the WHO’s Strategic Advisory Group of Experts on Immunization (SAGE) (10) established a Working Group dealing with vaccine hesitancy in 2012. “Vaccine hesitancy” is defined as a “delay in acceptance or refusal of vaccination despite availability of vaccination services” (11). This understanding acknowledges that vaccine hesitancy is a complex and context-specific issue, with a range of attitudes from complete vaccine acceptance to absolute vaccine refusal. It varies across time, place, and vaccines (11). It is not always a conscious decision. Sometimes, people are hesitant to get vaccinated because they simply do not fully understand the benefits of vaccines or the risks of vaccine-preventable diseases. WHO has considered this phenomenon to be one of the top 10 threats to global health and security since 2019 (12), and has also proposed the frameworks for measuring and testing strategies to address vaccine hesitancy (13, 14). In vaccination research, leading voices have urged to expand qualitative and quantitative investigations into public attitudes to inform vaccine confidence efforts (15–18).

China has made significant progress in reducing the incidence of HBV infection over the years. These achievements can be attributed to high universal vaccination coverage among children and the widespread administration of timely birth dose (TBD) vaccines to prevent mother-to-child transmission of HBV (19), both of which have exceeded 95% coverage rates. However, there are limited studies available that specifically address how to effectively tackle vaccine hesitancy while expanding the administration of the HepB in China.

It is important to note that vaccine hesitancy is a common phenomenon among those people who have concerns about the risks of the vaccine or lack of understanding of the benefits of the vaccine. Continuous improvement of the vaccination system is key to effectively tackling vaccine hesitancy and increasing HepB vaccination coverage in China. This includes providing accurate information about the vaccine and its benefits. Moreover, it is crucial for China to focus on focus on raising awareness, addressing misconceptions and establishing a comprehensive and effective immunization program and health system to encourage people to receive vaccination, as well as to ensure that individuals receive a sufficient dose of the vaccine.

This study endeavor aims to summarize how the Chinese government can take actions and promote vaccination through the National Hepatitis B Immunization Program. We conducted a comprehensive analysis by summarizing policy documents, reports, and scientific papers, thereby capturing the historical trajectory of the immunization program and drawing lessons. This research could contribute towards the identification of effective strategies to tackle vaccine hesitancy, the realization of universal health coverage for vaccines and the successful attainment of the goals set for 2030.

## 2 History of National Hepatitis B Immunization Program in China

As a vertically integrated program within the health system, the National Immunization Program (NIP) is responsible for delivering immunization services to children (20). China’s NIP has successfully controlled vaccine-preventable diseases over the past four decades.

The NIP is funded by various administrative levels of the government, and the financing strategy has been evolving since the 1970s. The vaccines in the NIP are available to all eligible recipients for free. In 1974, the Expanded Program on Immunization (EPI) was launched by WHO to tackle vaccine-preventable diseases and ensure that children from all countries could benefit from life-saving vaccines (21). In response to the WHO’s call for Member States to establish NIP that included a minimum set of recommended vaccines (22), the Ministry of Health of China established the Planned Immunization Program in 1978. The program was officially recognized and renamed as the NIP in 2002. Between 2002 and 2008, the NIP was expanded to include 15 vaccines to effectively combat and prevent vaccine-preventable diseases. This expansion was a major milestone in China’s efforts to improve child health and protect its population from preventable diseases (23).

National hepatitis B serosurveys have been conducted in China every 5–10 years since 1979. To date, four national HBV prevalence data sets have been published, in 1979, 1992, 2006, and 2014. The most recent serosurvey, conducted in 2020, also included data on hepatitis C (6).

China’s hepatitis B immunization strategy was initiated in 1985 with licensure of plasma-derived HepB. In 1983, Beijing Tiantan Biological Products Co., Ltd. successfully developed a plasma derived HepB, which was manufactured on a large scale and launched in 1985. The Ministry of Health of China first recommended routine vaccination of infants with HepB in 1992, with the first dose to be administered within 24 h of birth and subsequent doses at ages 1 and 6 months (24). Prior to 2002, infant vaccination in China was highly concentrated in large cities of the wealthier eastern provinces due to a number of factors, including high vaccine prices and user fees charged to parents by local health departments for vaccine purchase and administration (25). These factors made it difficult for families in rural areas and less wealthy provinces to afford to vaccinate their children.

**Figure 1:**
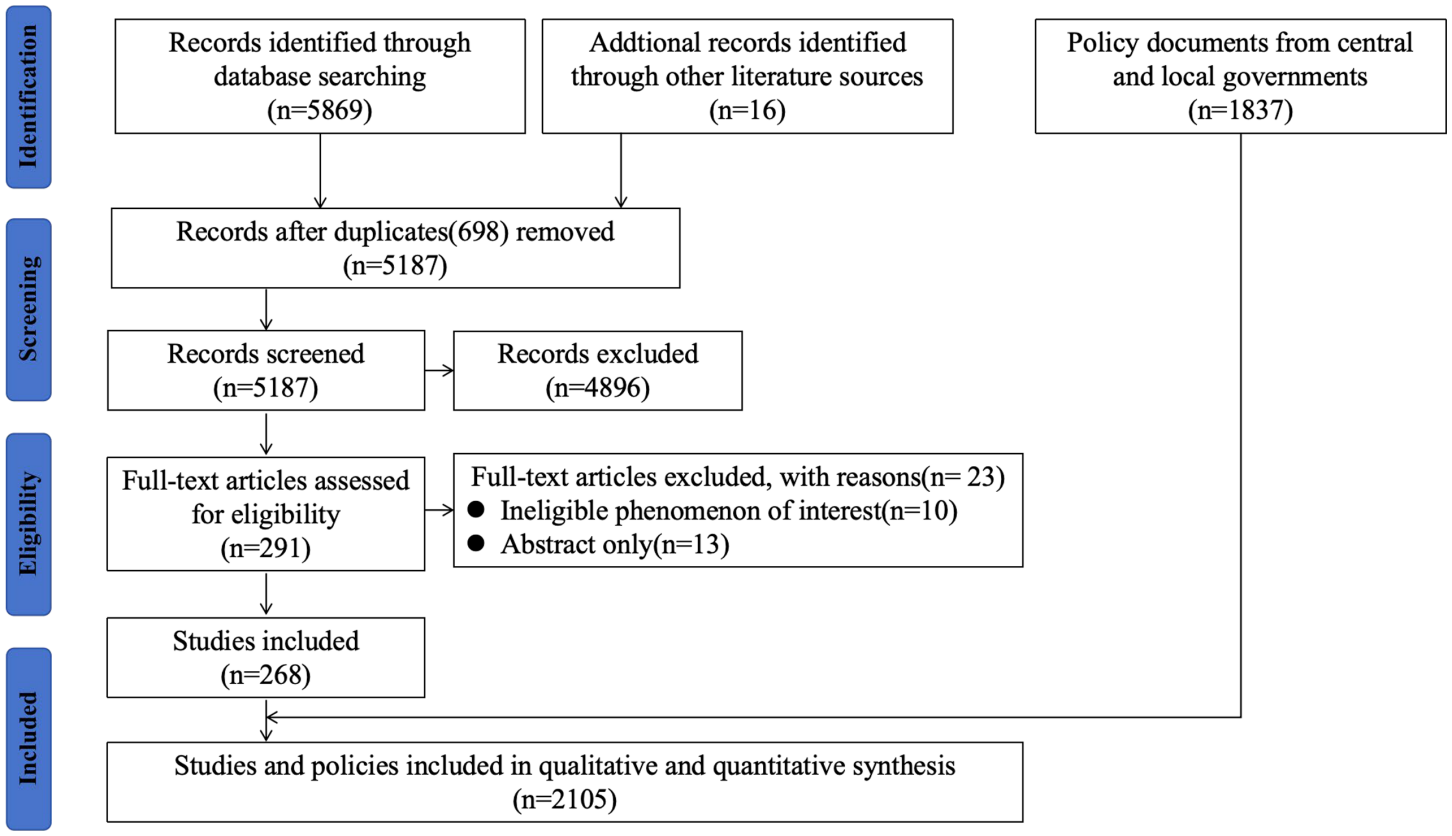
Review flowchart.

In 2002, China took a significant step by integrating the HepB into the national EPI program to make vaccines more affordable and accessible. This integration made the vaccine available at no cost to children up to 14 years of age. As part of this initiative, all newborns were required to complete the HBV vaccination without any fees. However, administration fees for HBV vaccinations, amounting to up to $1.10 per dose, was continued to be charged to parents. In the same year, the Ministry of Health of China began a project in collaboration with the Global Alliance for Vaccines and Immunization (GAVI) to ensure HepB availability in China’s poorest provinces and counties. The China-GAVI Project targeted the western provinces and the poverty-affected counties in the central provinces as its primary target areas. Over a span of 5 years, the China-GAVI project provided free HepB vaccines to approximately 5.6 million children born each year in 12 western provinces and in government-designated poor counties in 10 middle provinces. This initiative covered approximately 36% of China’s child population (25). By providing free HepB, the China-GAVI Project aimed to overcome financial barriers and improve the health of millions of children in China. This helped to increase vaccination coverage in these areas, which contributed to the overall goal of hepatitis B prevention and control in China. This project also contributed to build capacity in the Chinese health system to deliver vaccines, which has been used to support the implementation of other vaccination programs in China (see Figure 1).

**Figure 2:**
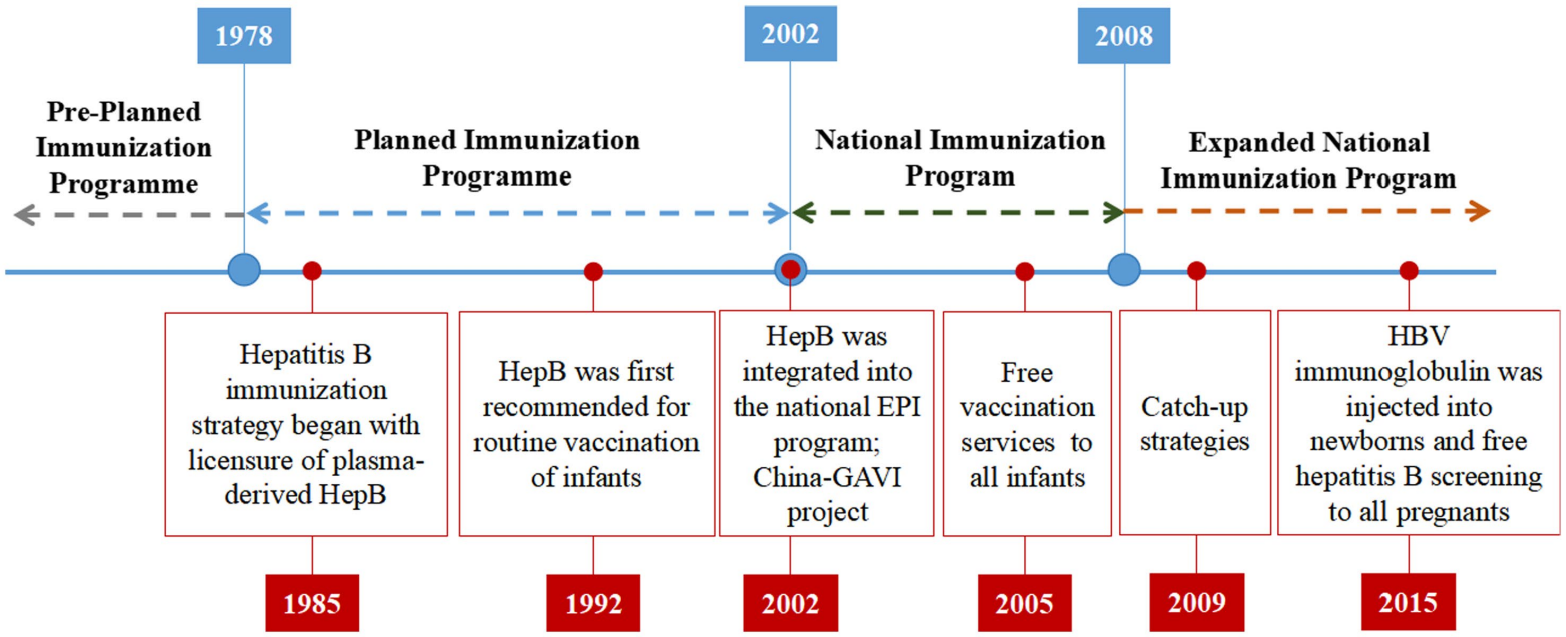
Timeline of China’s National Immunization Program and key events related to HepB.

In 2005, the State Council issued the “*Regulation on Management of Vaccine Circulation and Preventive*,” free HepB services provided to all infants with user fees eliminated provide favorable conditions for infants to receive vaccination for free, providing equal opportunity for all socioeconomic classes. This service is aimed at protecting infants from hepatitis B virus infection, which can cause liver damage and cancer risk. It is beneficial to infants who have been exposed to the virus and who need to receive repeated vaccinations. The service also provides an opportunity to reduce the incidence of hepatitis B infection and improve the overall health of the community. Therefore, from 2009 to 2011, for children <15 years of age who were born between 1994 and 2001 with incomplete, none, or unknown HBV vaccination history, the Ministry of Health of China proposed the catch-up strategies (26) based on effectively implementing a neonatal HBV immunization plan. Children who had received one or two doses of the HepB were revaccinated with two doses of the vaccine. Children who had not received any doses of the HepB were given HBV immunoglobulin, which is a blood product that contains antibodies against hepatitis B. It can help to protect against HBV infection if it is given soon after exposure to the virus. Children who had not received any doses of the HepB were also given three doses of the vaccine to get active immunization. The catch-up strategies were successful in increasing hepatitis B vaccination coverage among children in China, almost all children in this age group were protected against HBV infection. In 2015, HBV immunoglobulin was injected into newborns within 24 h after birth. This is because HBV immunoglobulin can help to block the virus from infecting the baby. Additionally, free hepatitis B screening was provided to all pregnant women as part of the program. Figure 2 provides an overview of China’s NIP and highlights key events related to the HepB.

Following the implementation of the China’s National Hepatitis B Immunization Program, the HBsAg seroprevalence rate for the population aged 1–59 years declined from 9.8% in 1992 to 7.2% in 2006, and for children age 1–4 years it was 0.96% (2). This represent a significant decrease in the prevalence of hepatitis B infection in China. In 2015, China achieved a children’s hepatitis B vaccination coverage rate of 99.6%, surpassing the WHO’s target of 90% (2, 3). This was a remarkable achievement and showed the commitment of the Chinese government to protecting the health of its children. The program shows that it is possible to achieve high vaccination coverage and reduce the burden of hepatitis B through strong political commitment, free vaccines, effective implementation, and community engagement.

## 3 Strategies to address vaccine hesitancy and promote vaccination in China

Despite the overwhelming evidence of the effectiveness and accessibility of vaccines, vaccine hesitancy remains a significant concern. Vaccine hesitancy is a complex decision-making process influenced by various factors, including personal experiences, risk perceptions, cultural beliefs, and confidence in authorities and medicine (9). When it comes to vaccine hesitancy, individuals may draw upon their own experiences, both positive and negative, with vaccines or healthcare systems. People may be hesitant to vaccinate if they or someone they know has had a negative experience with a vaccine, such as an allergic reaction. These experiences can shape their attitudes and beliefs towards vaccination. Furthermore, the risks of vaccines or underestimate the risks of vaccine-preventable diseases might be overestimated. Cultural beliefs also may influence populations’ views on vaccines. For example, some cultures may believe that vaccines are unnatural or that they can interfere with a person’s spiritual well-being. Confidence in authorities and medicine is another critical factor. Individuals’ trust in healthcare providers, public health authorities, and vaccine regulatory bodies can greatly impact their willingness to accept vaccines.

**Figure 3:**
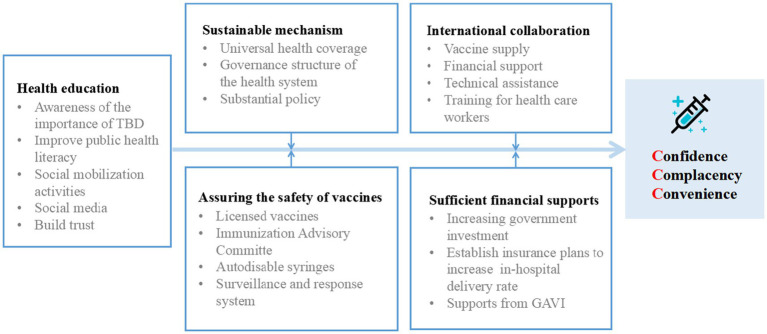
Strategies to address vaccine hesitancy based on the “3Cs” Model.

It is important to address the underlying reasons for vaccine hesitancy in order to increase vaccination rates. In 2014, the SAGE Working Group developed a “3Cs” Model of Vaccine Hesitancy to map three main factors that influence vaccine uptake (11). Vaccine hesitancy can be categorized into three main reasons: lack of confidence, complacency, and lack of convenience. Lack of confidence often stems from the inconsistent or incomplete information available about vaccines. Controversial debates surrounding vaccine effectiveness, safety, and potential side effects can lead to doubts and uncertainties among individuals. Inadequate communication or misinformation about vaccines further contribute to the lack of confidence in their benefits and safety. Complacency arises when individuals perceive the risk of vaccine-preventable diseases as low, especially considering the widespread vaccination coverage in the population. Some individuals may underestimate the severity of these diseases due to the success of vaccination programs, leading to a sense of complacency and a belief that vaccination is unnecessary. Lack of convenience is another significant factor contributing to vaccine hesitancy. Accessibility and availability of vaccination services play a crucial role in individuals’ decision to get vaccinated. Challenges such as limited access to healthcare facilities, inconvenient vaccination schedules, or language and cultural barriers can hinder individuals from receiving timely and appropriate vaccinations. The Chinese government addresses the convenience factors of the “3Cs” Model of vaccine hesitancy model by focusing on optimizing the affordability of vaccines by providing them free of charge or at low cost; ensuring the accessibility of vaccines by making them available in convenient locations, such as schools and community health centers; improving health literacy (27) by providing accurate information about vaccines to the public; and delivering vaccines in a culturally appropriate manner by taking into account the cultural beliefs and practices of the people being vaccinated. These efforts aim to promote vaccination among the population (Figure 3).

### 3.1 Establishing the sustainable mechanism

Ensuring everybody has access to quality health care is one of the most pressing priorities for the governments everywhere. Universal health coverage is a system that ensures that everyone has access to quality, affordable health care, regardless of their income or employment status. It is the goal of health care systems whereby all people, regardless of their economic conditions, receive the health services they need, which are of sufficient quality to provide a significant impact on their well-being, and thus reduce the risk of illness and disability for those who do not have access. Establishing a mechanism for internal coordination among multiple government entities and promoting international cooperation are crucial steps in improving vaccine coverage and developing sustainable infrastructure for health equity and justice. It is essential to employ comprehensive strategies in achieving these goals.

The National Hepatitis B Immunization Program is an integral part of China’s health system and is overseen by the National Health Commission. The program operates within a management network that aligns with the governance structure of the health system. Horizontally, the management network involves various entities that collaborate to supervise and manage the implementation of the NIP. These entities include health administrative departments (health commissions), technical institutions such as the China Centers for Disease Control and Prevention, service delivery institutions such as vaccine clinics hosted by designated qualified health facilities, and regulators such as medical products administrations and former food and drug administration (20). This collaborative approach ensures coordinated efforts and effective management of the program. Vertically, these governance units are established at multiple administrative governmental levels, depending on their respective functions within the program (8). This vertical integration allows for efficient coordination and implementation of the program across different levels of government. The National Hepatitis B Immunization Program is committed to providing safe, efficient, and effective vaccination services. Substantial progress has been made in various areas, such as governance, financing, service delivery, workforce, and information system development. These significant advances have contributed significantly to the overall success and efficiency of the program in preventing hepatitis B, and ensuring the well-being of the population. These achievements have not only been the result of the efforts and dedication of the program administrators, but also the result of the continuous efforts of our partners in the field to promote the hepatitis B prevention and control program.

**Figure 4:**
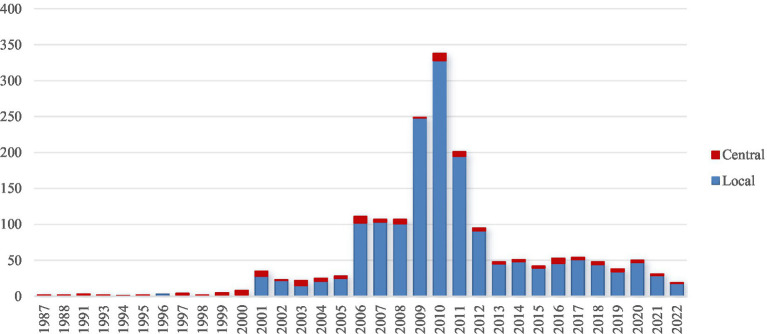
Number of central and local policy documents on Hepatitis B Immunization Program over years.

China has made universal vaccination for infants and children a priority. The government has developed various substantial policies (Figure 4) to promote and implement and simplify the vaccination process through streamlined appointment systems and reducing waiting times (28).

### 3.2 Assuring the safety of vaccines by surveillance and response

Concerns about the safety of the vaccine could contribute to vaccine hesitancy (29). One challenge is that the quality of vaccines in China can vary. Some vaccines are produced by domestic manufacturers, while others are imported. There have been reports of substandard vaccines being produced in China, which has raised concerns about the safety of vaccines. To address these concerns, it is important to provide accurate and evidence-based information about vaccine safety. Transparent communication from trusted healthcare authorities can help dispel misconceptions and alleviate fears. Additionally, highlighting the rigorous testing and monitoring processes that vaccines undergo before being approved for use can help build confidence in their safety.

In China, only licensed vaccines are utilized. Before receiving licensure, vaccines undergo rigorous evaluation for safety and efficacy through controlled clinical trials. The Experts Advisory Committee on Immunization Program (EACIP) (30, 31) of China was founded in 1982, and evolved into the National Immunization Advisory Committee in 2017 (31). The current panel of experts in the field of immunization and related areas consists of 33 individuals. These experts are carefully selected based on their knowledge, expertise, and experience in immunization practices and the control of vaccine-preventable diseases. The members of the EACIP are nominated and appointed by the Ministry of Health. The membership selection criteria for the Workshop are as follows: (i) expertise in research and development of vaccines: the members should have a deep understanding of vaccine research, development, and approval processes; (ii) testing and approval of vaccines: members should be able to perform the testing and approval process of vaccines, including the development of vaccine requirements and protocols, and the approval of vaccines by the authorities; (iii) pediatrics, infectious diseases, immunology, and management of health policy: members should be experts in the fields of pediatrics, infectious diseases, immunology, and health policy and have a strong background in these fields; (iv) public health, epidemiology and statistics: members should be proficient in public health, epidemiology, and statistics and have strong analytical skills; (v) ethics and health law: members should be ethical and responsible in their work and have a clear understanding of health law and regulations (30). Additionally, between 1999 and 2000, China conducted multiple assessments focused on injection safety, particularly in relation to vaccination. These assessments aimed to ensure the safe administration of vaccines and prevent any potential risks associated with injections (32). China has also improved its screening and diagnosis of HBV infection, so that people who are infected can be treated early and prevent the development of chronic disease.

Promotion of safe injection practices has also been important for chronic HBV prevention. In 2000, China banned the reuse of single-use medical devices. This regulation aimed to enhance patient safety and prevent the transmission of infections through contaminated medical equipment. In 2005, the Chinese Medical Association published guidelines for injections and other skin-piercing procedures, which provided healthcare professionals with standardized protocols and best practices to ensure safe and hygienic practices during such procedures. To support the implementation of safe injection practices, in 2007, auto-disable syringes became available for vaccine injections (33), which are designed for single use only, as they automatically disable after a single injection. This feature helps to prevent the reuse of syringes and reduces the risk of cross-contamination. By 2010, China successfully eliminated the use of reusable injection equipment and transitioned to the universal use of disposable and auto-disable syringes. This significant shift in injection practices further enhanced patient safety.

Importantly, the *Vaccines Administration Law* (34) was implemented in 2019, marking the first comprehensive and vaccine-specific law in China’s history. It establishes a legal framework aimed at enhancing vaccine administration, supply, and quality. In addition to the aforementioned measures, China also prioritizes research, development, and manufacturing of vaccines as part of its comprehensive approach to immunization. The government recognizes the importance of advancing vaccine technologies, improving vaccine efficacy and safety, and expanding the vaccine portfolio to address emerging infectious diseases. Through supporting scientific research, clinical trials, and collaborations with international partners to develop innovative vaccines, China has enhanced its vaccine capabilities and contribute to global efforts in combating various diseases. Furthermore, China places a strong emphasis on maintaining high standards for vaccine service delivery. Notably, the *Vaccines Administration Law* includes provisions to maximize vaccine protection among children (34). It mandates that the ministries of health and education collaborate to develop guidelines for checking vaccination records of school children. This approach ensures that children who are new to a school and are in need of one or more doses of vaccine can be referred to their designated immunization clinic to receive the necessary vaccinations. When a child enrolls in a new school, their immunization records are typically reviewed to determine if they are up to date with the required vaccinations. If it is found that the child requires one or more doses of vaccine, the school can facilitate the referral process by providing the necessary information to the immunization clinic. The immunization clinic, in turn, can then schedule an appointment for the child to receive the required vaccinations. This ensures that the child receives the necessary vaccines in a timely manner, helping to protect their health and prevent the spread of vaccine-preventable diseases within the school community. In China, vaccines are also monitored for safety and effectiveness during their use. Adverse events following immunization are reported to a national monitoring system to ensure the ongoing safety and effectiveness of vaccines. It helps to maintain public confidence in vaccines and ensures that any potential safety concerns are addressed in a timely and effective manner.

### 3.3 Providing sufficient financial supports

Reducing financial barriers by providing affordable or free vaccination services is necessary. The government of China regards health equity as a fundamental principle of social justice and fairness, while funding-related issues were the main barriers to improve the coverage of the birth dose (35). In line with this commitment, the Chinese government has implemented various policies and initiatives to address health disparities and promote health equity. These efforts aim to reduce inequalities in access to healthcare, improve healthcare infrastructure in underserved areas, and enhance the overall health outcomes of marginalized populations. The cost of vaccination is one of the most common reasons why people do not get vaccinated. Vaccines can be expensive, especially in developing countries. In some cases, the cost of vaccination can be a barrier to access, even for people who have health insurance. One is to subsidize the cost of vaccines, either through government programs or through private donors. Another is to make vaccines more affordable by producing them in bulk and negotiating lower prices with manufacturers. With the increasing government investment, HepB has been integrated into the NIP in process since 2002, now it is free to all, which improves the immunization coverage. Despite these efforts, the cost of vaccination can still be a barrier to access for some people in China. The government is continuing to work to make vaccines more affordable and accessible to everyone.

The majority of chronic HBV infections are a result of transmission of HBV from mother to child before HepB is available (36). Before the introduction of the HepB, the primary mode of HBV transmission was from an infected mother to her newborn during childbirth. This vertical transmission was a main contributor to the high prevalence of chronic HBV infections worldwide. Birth-dose vaccination is a key intervention for prevention of HBV infection in infants. The vaccine stimulates the immune system to produce protective antibodies, preventing HBV infection in the newborns and reducing the likelihood of chronic infection. So, providing subsidies to parents to encourage delivery of infants in hospitals is one way to improve hospital delivery rates. The government established insurance plans to ensure access to healthcare and birth facilities, especially in impoverished, remote, or ethnic minority areas. By taking these steps, the government can help to improve hospital delivery rates and ensure that all women have access to safe and quality care during childbirth. In 2000, the Chinese government took significant steps to address maternal mortality rates and eliminate maternal/neonatal tetanus by implementing a comprehensive program (23). This program involved collaboration between the Ministries of Health and Finance, as well as the State Council. It aimed to ensure that all women in China have access to quality maternal healthcare services and receive appropriate medical care during pregnancy, delivery, and the postpartum period. The principle “whoever delivers the baby vaccinates the baby” (3, 24) is a simple but effective way to ensure that all infants born in birthing facilities receive a birth dose of HepB. The increase in the in-hospital delivery rate from 44% in 1985 to 99% in 2013 is a significant achievement in improving access to quality healthcare services during childbirth in China (3). This substantial increase reflects successful efforts to promote hospital deliveries and ensure the safety and well-being of both mothers and newborns (3).

Besides, the global vaccine alliance GAVI provides immunization support to 76 resource-limited countries (37). Since 2002, GAVI has worked closely with rural and western areas of China to promote the birth-dose policy for immunization, ensuring that newborns in these regions have access to timely and essential vaccinations (3). The China-GAVI project was funded equally by GAVI and the government of China. Healthcare workers at healthcare facilities, mostly at the township hospitals level and in community centers, participated in the project (25). This support includes funding for the procurement of vaccines, strengthening healthcare systems, and improving vaccine delivery infrastructure. Starting from 2021, GAVI has provided subsidized support to HBV birth-dose vaccines, conditioned on their replenishment. This change has been widely welcomed by the liver health community as it is expected to significantly increase infant vaccination coverage by up to 80%, from only 38% in 2015 (2). In addition, GAVI projects subsidize providers as an incentive for providing TBD. Overall, China has attained the goals of increasing infant birth coverage, and the efforts are continuing to further advance its progress.

### 3.4 Collaborating with international organizations

The collaboration and support from organizations such as Merck and GAVI have indeed made significant contributions to the hepatitis immunization program. Their involvement has helped in various ways, including vaccine supply, research and development, financial support, and technical assistance. This support includes training healthcare workers, developing vaccination strategies, and monitoring program performance.

Vaccines often suffer from underinvestment (38). Efficient utilization of vaccines is vital to maximize their impact. This includes establishing robust immunization systems, ensuring the availability of vaccines, training healthcare workers on proper administration and storage, and implementing effective monitoring and surveillance systems. Regular evaluation and assessment of vaccination programs can help identify areas for improvement and optimize vaccine utilization. By addressing affordability, accessibility, trust, and efficient utilization, governments and vaccine purchasers can effectively implement HepB vaccination programs. This comprehensive approach is essential to ensure that new vaccines, including HepB, reach the populations that need them most and contribute to the prevention and control of vaccine-preventable diseases (39). Merck, as a pharmaceutical company, plays a crucial role in manufacturing and supplying hepatitis vaccines, ensuring a steady and reliable vaccine supply for immunization programs. Because of the potential risk of transmission of live HBV and other blood-borne pathogen, as well as the limited number of blood donors in China, the country has implemented a policy of universal hepatitis B vaccination. In 1989, when Dr. Vagelos served as the chairman and CEO of Merck, he made a decision to sell the technology of producing the HepB to China for $7 million, aiming to help the country fight against its biggest threat to public health. In this project, Merck received no profits or royalties from the sale (40). Beijing Tiantan and Shenzhen Kangtai Biological Products Co., Ltd. introduced the production technology of recombinant DNA HepB from Merck in 1992, received the drug certificate and launched the products in 1995. This helps in enhancing the effectiveness and accessibility of HepB. In collaboration with GAVI, China has increase the awareness of the importance of TBD among providers and parents, intensified training for health care workers, monitored and supervised vaccination activities, built collaboration between delivery service (maternal and child health) and vaccination service and subsidized providers as an incentive for providing TBD (25).

The collaboration and support from Merck and GAVI, along with other stakeholders, have been crucial in advancing the hepatitis immunization program, ensuring access to vaccines, and working towards the goal of eliminating hepatitis B as a public health threat. They are also committed to working with other stakeholders, such as non-governmental organizations and the private sector, to promote health equity. Governments should prioritize maximizing communication and collaboration to ensure that equity goals are central to all aspects of vaccine campaigns (41).

### 3.5 Strengthening health education on immunization

One common barrier to vaccination programs is the lack of knowledge regarding the benefits of vaccination and awareness about the importance of vaccination (18). Many people are not aware of the benefits of vaccination or the risks of vaccine-preventable diseases. This lack of awareness can lead to parents choosing not to vaccinate their children. The loss of vaccine confidence has indeed been associated with declining HepB vaccination coverage, particularly during the COVID-19 pandemic. Vaccine confidence refers to the trust and belief individuals have in the safety and effectiveness of vaccines (42, 43). Research has shown a significant level of HepB hesitancy among expectant mothers, which is associated with factors such as marital status, educational attainment, HBV-specific knowledge, perception, and behavioral skills. To address this, targeted health education is necessary, particularly for married women with lower educational attainment, who are vulnerable populations, in order to enhance their knowledge and shape their perception and behavioral skills towards a higher acceptance of hepatitis B vaccination. Research findings have indeed highlighted a significant level of HepB hesitancy among expectant mothers. This hesitancy is influenced by various factors, including marital status, educational attainment, HBV-specific knowledge, perception, and behavioral skills. To effectively address this issue, targeted health education is crucial, particularly for married women with lower educational attainment, as they are considered vulnerable populations (44). The China-GAVI project developed information, educational materials, and communication resources and conducted a number of social mobilization activities in 22 provinces, including public advocacy, media interactions, posters, and banners, to provide information to the public (45).

Social media platforms have become a critical tool for understanding public sentiment on vaccination and its impact on the public health system. The importance of social media platforms has been recognized as a crucial factor in infodemic (46) and public sentiment on vaccination (47). Internet-based and social media technologies, allowed widespread access to information, as well as misinformation, and fueled the viral spread of questioning about vaccines (48). The analysis of social media data has been proved to be an effective way to understand public sentiment on vaccination. However, the traditional methods for analyzing the public sentiment on vaccination are not efficient enough to capture the complexity of the issue. The use of AI technology, such as text mining, has also proved to be an effective way to analyze the public sentiment on vaccination The source of information considered by parents plays a fundamental role in the decision-making process regarding childhood vaccinations, as it can greatly influence their acceptance or refusal of vaccines. Parents rely on various sources of information to gather knowledge about vaccines, including healthcare providers, family and friends, the internet, social media, and community networks. The credibility, accuracy, and trustworthiness of these sources significantly impact parents’ perceptions and attitudes towards vaccination. In December 2013, widespread media reports of the circumstances of the infant deaths that followed hepatitis B vaccination (49). Seventeen deaths and one case of anaphylactic shock were reported following HBV vaccination. As a precautionary measure, the use of HepB from the implicated company was suspended during the investigation. Experts thoroughly assess the safety, efficacy, and quality of the HepB vaccines produced by the company in question. This includes evaluating manufacturing processes, conducting laboratory tests, and reviewing clinical data. The goal is to determine whether there are any concerns or risks associated with the vaccines and to take appropriate actions based on the findings. However, subsequent findings indicated that the deaths were not caused by the hepatitis B vaccine, and its use was resumed. Before the event, 85% of respondents regarded domestic vaccines as safe, but this decreased to 26.7% during the event. At the height of the crisis, 30% of parents reported being hesitant to vaccinate and 18.4% reported they would refuse HepB (49).

With the timely and credible investigation, combined with the proactive outreach to stakeholders and the media, it is possible to mitigate the negative impact of future coincidental adverse events following immunization. Rebuilding a trust relationship between individuals and healthcare providers is of utmost importance. Clear and transparent communication is key in mitigating the negative impact of adverse events. Providing regular updates on the investigation process, sharing the findings, and addressing any safety concerns can help build trust and maintain confidence in vaccination programs. Open channels of communication, such as hotlines or dedicated websites, can also allow individuals to seek information and have their questions answered. Additionally, proactive measures should be taken to educate the public about the concept of coincidental adverse events following immunization. Understanding that adverse events can occur independently of the vaccine and that thorough investigations are conducted can help alleviate concerns and prevent vaccine hesitancy. This can be achieved by actively engaging with healthcare providers and community leaders to promote accurate information, address misinformation, and foster open communication. Moreover, harnessing the power of social influences and trusted figures can play a pivotal role in endorsing vaccination and reinforcing the critical significance of immunization.

## 4 Conclusion

Regarding vaccine hesitancy in China, it is important to note that the Chinese government has been actively working to address this issue. They have implemented various strategies to improve vaccine confidence and promote vaccination. These efforts include strengthening public education campaigns, enhancing vaccine safety monitoring systems, and ensuring the availability and accessibility of vaccines. High vaccine coverage is facilitated by strong implementation of the National Hepatitis B Immunization Program. The urgent need to keep working with healthcare professionals and the public on eliminating vaccine hesitancy is essential, as it can be a significant impediment to implementing one of the most effective tools we have for HBV control worldwide. It is crucial to promote the public awareness of HBV vaccination, and to leverage the power of social media to provide information and guidance to those who are hesitant to get vaccinated or not yet vaccinated. We must foster a positive environment for healthcare professionals to provide information and support to those who are hesitant to get vaccinated, and to reinforce the importance of vaccination for preventing and treating HBV. Furthermore, it is crucial to ensure that healthcare professionals are well-informed about the available vaccines and equipped to provide accurate and reliable information to individuals who may be hesitant to receive vaccination or who have already received their initial dose (50). This will empower healthcare providers to address concerns, dispel myths, and offer appropriate guidance, ultimately fostering vaccine confidence and uptake.

In terms of future plans until 2030, especially realizing the goals of Healthy China Action Plan, it is expected that China will continue to prioritize efforts to combat vaccine hesitancy. This may involve further strengthening public awareness campaigns, providing accurate and transparent information about vaccines, addressing concerns and misconceptions, and building trust in the healthcare system. We need to take interventions to build confidence on vaccines, increase acceptance, and promote adequate immunization behaviors. Based on targets of the WHO’s *Global health sector strategy on viral hepatitis 2016–2021*, we highlight further priorities for action. Firstly, government officials and partners need to assess the suitability of various vaccines for the respective health systems and populations—for example, in terms of availability, affordability, efficacy, and dosing and storage requirements. Secondly, safe and effective vaccines and tools should be supplied. Thirdly, international collaboration provides technical expertise, financial supports and guidance to governments and health authorities in implementing effective hepatitis immunization programs. Chinese government also has been committed to increasing infant healthcare coverage. Due to the misinformation can prevent vulnerable populations from getting safe and accurate healthcare. To ensure equal opportunity for all infants, including those born at home and in remote areas, specific strategies can be implemented to facilitate their access to HepB. Meanwhile, to encourage parents who may be hesitant about vaccines, it is crucial to focus on building trust and promoting protective behaviors through health education. This approach is highly effective in preventing the transmission of HBV from mother to child. By providing accurate information, addressing concerns, and emphasizing the importance of vaccination, we can help parents make informed decisions and take necessary steps to protect their children from HBV.

China has already achieved significant progress in vaccination coverage and disease prevention through its vaccination programs. This study summarized the achievements and lessons of China’s National Hepatitis B Immunization Program and strategies to overcoming vaccine hesitancy, future research should continue to estimate vaccine hesitancy among the vulnerable populations and explore implementation research on promoting vaccination. These strategies, when implemented effectively and continuously, can help address vaccine hesitancy, improve vaccine acceptance and coverage rates, and achieve SDGs by 2030.

## Author contributions

HJ: Conceptualization, Formal analysis, Investigation, Methodology, Resources, Software, Visualization, Writing – original draft, Writing – review & editing. CW: Supervision, Writing – review & editing, Methodology.
